# Overt visual attention and between-limb asynchrony for bimanual reaching movements

**DOI:** 10.1007/s00221-023-06552-6

**Published:** 2023-01-19

**Authors:** S. D. Sardar, S.-H. Yeo, J. E. Allsop, T. D. Punt

**Affiliations:** 1 School of Sport, Exercise and Rehabilitation Sciences, The University of Birmingham, Edgbaston, Birmingham, B15 2TT UK; 2 Central Flying School, RAF College Cranwell, Sleaford, NG34 8HB UK

**Keywords:** Movement, Bimanual coordination, Reaching, Eye-hand coordination, Coupling, Asynchrony

## Abstract

Although synchrony between the limbs is an often-cited feature of bimanual coordination, recent studies have also highlighted the small asynchronies that can occur. The visuo-motor demands of any bimanual task are considered central to the emergence of asynchrony, but the relationship between the two remains largely unexplored. This study aimed to address this issue. Hand and eye movements were measured in 19 participants, while they made either unimanual or bimanual reach-to-point (aiming) movements to targets presented on a touchscreen. Bimanual movements were either congruent (same-sized targets) or incongruent (different-sized targets). Resulting hand data showed many of the typical patterns of movement previously reported. While temporal coupling between the limbs remained largely evident for bimanual movements, small between-limb asynchronies were apparent and demonstrated clear associations with the competing precision requirements of the targets and related visual attention. Participants mainly directed their gaze towards the more difficult target with corresponding reaching movements demonstrating greater precision than for the easier target. Additionally, there was a reliable tendency for the hand reaching towards the more difficult target to lead. Importantly, it was the competing visuo-motor demands of individual movements rather than overall difficulty that resulted in greater between-limb asynchrony; accordingly, where both targets were small (i.e., the most difficult condition), asynchrony was significantly less pronounced than for incongruent bimanual conditions. The results show how the visuo-motor system balances its apparent drive for synchrony in coordinating bimanual movements with the competing demands that characterise the constituent unimanual movements.

## Introduction

Being able to use both hands in a coordinated manner is critical to performing everyday functions; for example, consider lifting a tray or using a knife and fork. This study is concerned with how humans use vision to control moving both upper limbs at the same time. Bimanual movements demonstrate a strong tendency to be coupled, even when tasks require each limb to make movements to targets that differ in size. However, as vision can only be directed towards one target at a time, such circumstances create a challenge for the individual as to where to direct vision as the movements unfold. This challenge may be considered to have a strong competitive element with each limb/target *competing* for visual resources.

For unimanual movements, factors influencing the so-called *index of difficulty* (IoD) relate to target features. Accordingly, it has long been known that target size and target distance have a systematic effect on movement time, as explained by Fitts’ Law (Fitts 1954). For bimanual movements to separate targets, this relationship is maintained where these are made to targets with the same IoD; however, where bimanual movements are made to targets with differing IoDs, the overriding influence exerted by coupling ensures that Fitts’ Law is violated. In such cases, the limb moving to the easier target tends to be slowed, so that movement starts and ends at the same time as the limb moving towards the more difficult target (Kelso et al. 1979; Jackson et al. 1999). Importantly, while these movements are ostensibly coupled, many studies have highlighted the small asynchronies that emerge and become more pronounced during the latter stages of movement. These asynchronies are thought to be driven, at least partly, by the relative difficulty of the limb movements involved (Fowler et al. 1991; Riek et al. 2003; Bruyn and Mason 2009; Hesse et al. 2010; Srinivasan and Martin 2010; Miller and Smyth 2012).

In support of the above, Miller and Smyth (2012) showed temporal synchrony to be enhanced in the absence of visual feedback (of both hands and targets). Furthermore, where visual fixation is constrained (e.g., where participants fixate a midpoint between two targets), synchrony is greater than in *free view* conditions (Hesse et al. 2010; Jackson et al. 2002), while precision is diminished (Bruyn and Mason 2009; Jackson et al. 2002). As Srinivasan and Martin (2010) suggest, “the relationship between gaze orientation and hand movements seems to reflect a trade-off between accuracy and synchronization” (pp. 403). While these studies have highlighted that asynchrony during bimanual activity is modulated by the task-related demands on vision, the nature of this relationship is not well understood.

As well as the demands of the task, performance may also be modulated by the relative skill of the limbs involved. For unimanual movements, an individual may be relatively fast and more accurate to perform the same movement with their dominant compared with their non-dominant limb (Roy et al. 1989). For bimanual movements, how individuals manage these *internal* issues while also managing the varying task demands (*external* issues) that affect movement difficulty remains relatively unexplored. For bimanual tasks where the target IoD is identical, one might expect attention to be directed towards the non-dominant (less skilled) limb. However, early research suggested that the opposite was true (Peters 1981). Subsequently, Honda (1982) had right-handed individuals make bimanual reaching movements to the same-sized targets and similarly found a strong tendency for initial rightward eye movements. More recent research, while not measuring eye movements, also strongly suggests a rightward bias of attention (for right-handers) during bimanual reaching movements (Buckingham and Carey 2009; Buckingham et al. 2011).

However, studies including a more detailed analysis of eye movements (i.e., overt attention) during bimanual tasks with right-handers found terminal eye movements (i.e. the direction that vision was directed at the time movements were completed) to be directed to the left when the IoD of targets was identical (Riek et al. 2003). In both these studies (Riek et al. 2003; Srinivasan and Martin 2010), task performance was constrained with zero tolerance for error; if participants did not hit the targets, trials were excluded and then repeated and no error data were reported. Such demanding requirements may have given rise to the ‘hover’ phase observed, with participants prioritising accuracy by visually checking the position of their limbs; the ‘hover’ phase refers to participants interrupting the movement of the leading limb just shy of its target until the other limb catches up, before completing the final part of both movements synchronously.

In summary, it remains unclear how visual resources are allocated to guide bimanual movements and how this relates to any resulting asynchrony between the limbs. Perhaps, one of the reasons for this is that surprisingly a few studies have attempted to measure eye movements during visually guided bimanual tasks.

The current study aimed to explore eye and hand movements, while individuals made unimanual and bimanual aiming movements to targets with either the same or different IoDs. We were interested in how target difficulty modulated inter-limb coupling and the use of visual resources. We were particularly interested in understanding the relationship between any asynchrony and eye movements (overt attention). Furthermore, while our bimanual aiming movement task provided challenging targets, we also measured limb movement precision (distance from target centre) providing a further indication of performance and how this related to asynchrony and eye movements.

## Methods

### Participants

Nineteen individuals (10 right-handed) aged between 18 and 23 years participated in the study as unpaid volunteers. All participants had normal vision and had no known neurological or musculo-skeletal disorders. Handedness was confirmed using the Edinburgh Handedness Inventory (Oldfield 1971).

The project was reviewed and approved by the University of Birmingham’s Science, Technology, Engineering and Mathematics Ethical Review Committee. Participants provided written informed consent prior to taking part.

### Apparatus and procedure

**Fig. 1:**
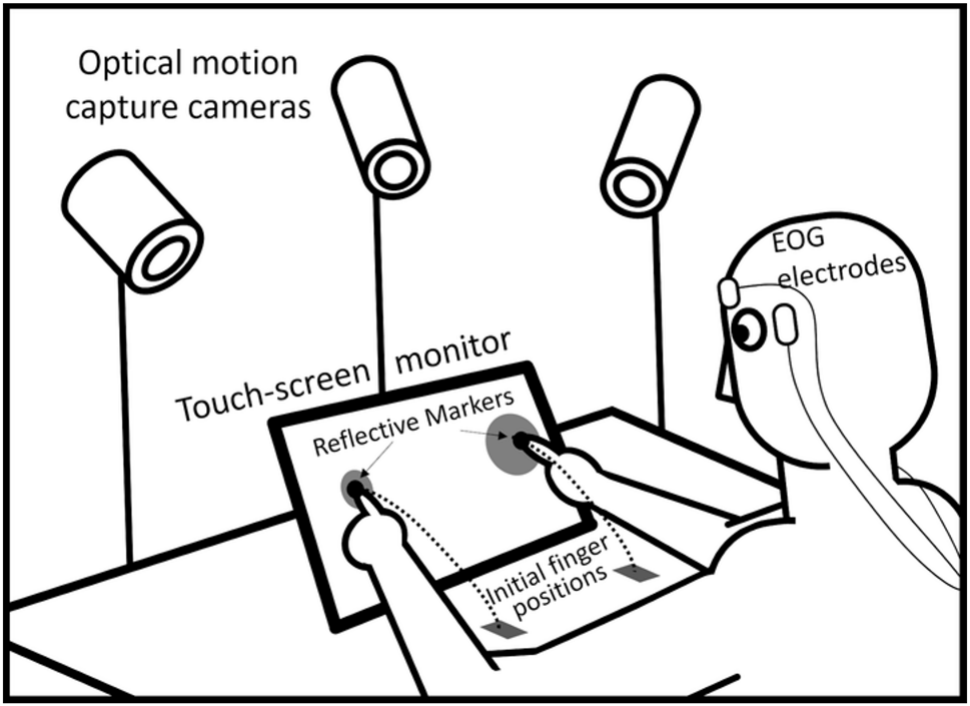
Schematic representation of the task. Unimanual and bimanual aiming movements were made to targets presented on either side of the mid-sagittal plane (see text for further details)

The experiment involved the measurement of hand and eye movements. Hand movements were recorded using a three-camera motion capture system (ProReflex, Qualisys AB, Gothenburg, Sweden) positioned around the workspace (see Fig. 1). A 5 mm reflective marker was attached to the nail of the index finger of each hand and was tracked by this system with a sampling rate of 200 Hz.

For analysis, markers were identified and distinguished from each other using a custom-written script in *MATLAB* (The MathWorks Inc., Natick, MA, USA). Although this function was automated, all trials were also visually examined to check accuracy. Data were filtered using a 5th-order low-pass Butterworth filter with a cut-off frequency of 20 Hz. *X*, *y,* and z position vectors were differentiated and then combined to yield absolute velocity, and this was then used to calculate the dependent variables.

Horizontal eye movements were recorded using electro-oculography (EOG). Self-adhesive surface electrodes were attached to the canthi of the left and right eye, in addition to a *ground* electrode in the centre of the forehead. EOG signals were sampled at 2000 Hz. These signals were amplified (2 K) and band-pass filtered (0.1–30 Hz) using an AC preamplifier (Grass Instruments LP122).

Timing and trial events were recorded continuously using an in-house programme (MATLAB) built around an Arduino UNO R3 Development Board and a data acquisition board (DAQ 2500, National Instruments Corporation).

Participants sat facing a 23-inch LCD touchscreen monitor (Dell S2340T) positioned on a table in their mid-sagittal plane; the screen was tilted backwards at an angle of 27° from vertical. The participants began each trial with their left and right index fingers resting on two small, roughened areas positioned 25 cm apart and 35 cm from the touchscreen monitor’s lowest edge; these served as the starting position for each trial. In response to targets appearing on the touchscreen monitor, they then made either unimanual or bimanual movements. Participants made left hand movements to targets presented on the left side of the screen and right-hand movements to targets appearing on the right side of the screen. Targets could be either small (diameter = 2 cm) or large (diameter = 10 cm), the latter having a lower IoD.

The target centre points were marked within the programme, on a 1920 resolution monitor; these appeared at the 300 pixel mark (for left target) and 1620 pixel mark (for right target) along the *X* axis and at 540 pixels along the *Y* axis. The centre of each target was therefore at a visual angle of approximately 7° from fixation.

**Fig. 2:**
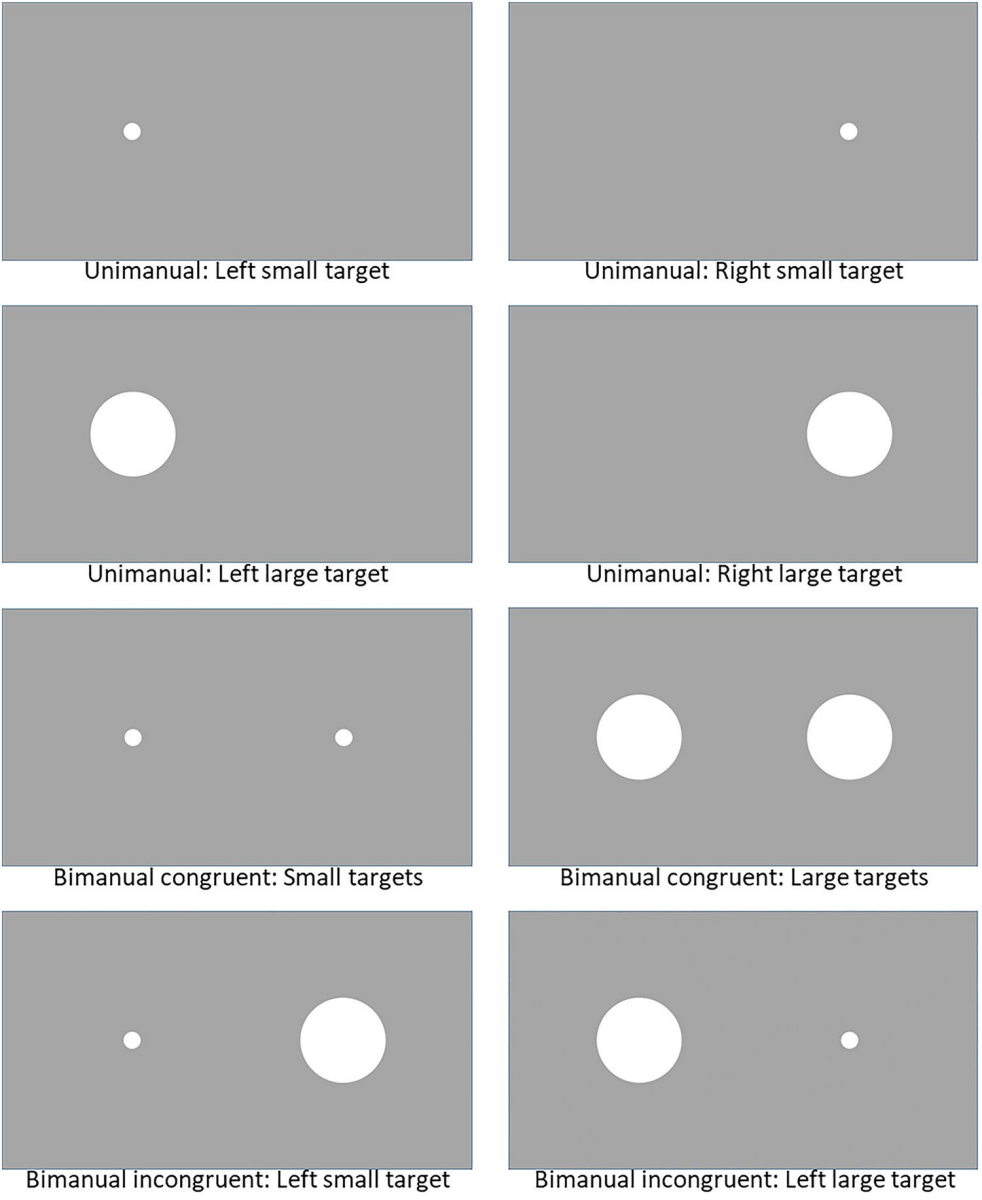
Visual stimuli used in experiment (see text for further details)

Where single targets appeared, unimanual movements were required, and where two targets appeared, bimanual movements were required. For bimanual trials, targets could be either congruent (i.e., the same size) or incongruent (i.e., different size). There were eight experimental conditions in total (see Fig. 2); four conditions required unimanual responses and four conditions required bimanual responses.

Targets on the left and right side of the screen were always an equal distance apart but were offset (in parallel) laterally and vertically on the screen at random by a maximum of 200 pixels on each trial (the maximum offset of 200 pixels equated to 5.29 cm). This was to ensure that each trial presented a visual challenge and that participants did not simply repeat exactly the same movement(s) on every trial. However, for bimanual trials, targets on the left and right side of the screen were always an equal distance apart, i.e., offsets were equal and occurred in parallel, laterally and vertically.

Each trial began with the participant’s hands in the starting position. A fixation cross was presented in the centre of the screen for a random period between 1000 and 3000 ms prior to the targets appearing. The fixation cross was removed as the targets were presented and the targets then remained on the screen for 3000 ms. Participants were instructed to reach and touch the centre of the targets as fast and as accurately as possible. When the screen was touched, the *touch-points* (superimposed on the targets) became visible for 1000 ms; this provided a degree of visual feedback for the participants once each trial was completed and they withdrew their fingers from the touchscreen. Following feedback, a blank screen appeared and each subsequent trial was manually triggered by the experimenter with a key press. A practice session (eight trials; one for each condition) preceded the experimental trials to allow participants to familiarize themselves with the task. There were 80 experimental trials per participant (10 for each condition) and condition was randomised across trials. As each trial was manually triggered by the experimenter, participants were able to take a break at any point during the experiment.

### Dependent variables

A series of performance measures were derived, based on data gathered from the motion capture system, EOG, and touchscreen. Details of how each measure was derived are provided below.

### Limb movement performance

(i) Reaction time (RT): time from target onset (touchscreen) to movement onset (defined as the first frame when hand speed exceeded 50 mm/s).
(ii) Movement time (MT): time from movement onset to movement end (touchscreen). Given previous reports of a *hover phase* towards the end of similar bimanual movements (Riek et al. 2003; Srinivasan and Martin 2010), limb speed following peak speed was examined but never fell below 50 mm/s before the touchscreen was touched.
(iii) Response time (ResT): time from target onset to movement end; i.e., (i) + (ii).
(iv) Acceleration time (AT): time from movement onset to the time peak speed (see below) reached.
(v) Peak speed (PS): the highest speed (in mm/s) reached during the limb’s movement towards the target.
(vi) Deceleration time (DT): time from the frame peak speed reached to movement end.
(vii) Target error (TE): the distance (in mm) from the centre of target circle to the touch point.

### Coupling relations between the limbs (relative synchrony)

For all bimanual conditions, the level of synchrony between the limbs was examined at three time-points: (i) *movement onset*; (ii) *time of peak speed*, and (iii) *movement end*.

We were interested in the ‘coupling’ between the limbs; this included understanding the absolute synchrony between the limbs as well as any lateralised bias that may have emerged. These were captured as:

(i) Signed lag—this captured the time (in ms) and the direction of any asynchrony; a negative lag represented a non-dominant hand lead while a positive lag represented a dominant hand lead.
(ii) Absolute lag: the time (in ms) separating the limbs regardless of direction.

### Eye movements

Analysis of EOG data focused on the proportion of time spent looking towards the dominant and non-dominant side as a proportion of Response Time. The proportion of time for which gaze was directed to each side was expressed as a value between − 1 (100% non-dominant) and + 1 (100% dominant).

### Statistical analysis

Mean data for each participant and for each condition were derived from the repetitions completed. For most DVs, a 3 × 2x2 analysis of variance (ANOVA) with repeated measures was conducted; factors (levels) were *Condition* (unimanual vs. bimanual congruent vs. bimanual incongruent), *Side* (non-dominant vs. dominant), and *Size* (small vs. large). Additionally, for bimanual movements, we examined synchrony at various time-points (see above) via a series of 2 × 2 × 2 ANOVAs with repeated measures; factors (levels) were *Congruence* (congruent vs. incongruent), Non-dominant size (small vs. large) and *Bias* (initial number vs. its additive inverse). The reason for the latter factor was to provide an indication of whether any mean asynchrony deviated reliably from synchrony, which would be indicated by zero. However, our approach provided a more conservative approach than simply comparing with zero. Similarly, for eye movement measures, the *Bias* factor allowed us to examine whether any directional bias in gaze direction differed reliably from the 50:50 (equal) situation, that would be indicated by zero. For all ANOVAs presented, statistical significance was set at *p* < 0.05 and resulting interactions were explored via simple effects; these analyses were conducted using Bonferroni adjustment. Estimates of effect size were reported via partial eta-squared.

## Results

### Limb movements

Table 1 provides mean (and standard error) values for limb movement measures and notes the significant main effects and interactions found for each. Across all these analyses, *Side* (dominant vs. non-dominant hand) did not feature as a significant main effect or in any interactions; data presented were therefore collapsed across this factor. Statistical analysis of several kinematic measures revealed a significant main effect of *Condition* (see Table 2 for related pairwise comparisons) and *Condition* x *Size* interaction (see Table 3 for the individual *Size* effects for each condition). These significant main effects and interactions largely replicate the data of previous bimanual movement studies and highlight two important previously established findings. First, data highlight the *cost* of undertaking bimanual compared with unimanual movements; bimanual movements were slower than unimanual movements (for RT, AT, PS, MT, DT, and ResT). Second, consistent with Fitts’ Law, movements to small targets were slower for unimanual and bimanual congruent movements (for RT, PS, MT, DT, and ResT); however, this was not the case for bimanual incongruent movements where RT, PS, and ResT were comparable for small and large targets. In addition, the component measures of MT and DT showed significantly shorter values for *small* targets.

**Table 1:** Mean (and standard error) values and significant effects as a function of *Condition* and *Size* for limb movement measures

	Unimanual		Bimanual congruent		Bimanual incongruent		Effects
	Small	Large	Small	Large	Small	Large	
Reaction time (ms)	445 (13)	419 (12)	491 (23)	448 (18)	491 (20)	481 (22)	Cond, size, cond*size
Acceleration time (ms)	159 (8)	154 (8)	175 (9)	170 (9)	176 (8)	175 (9)	Cond
Peak speed (mm/s)	1244 (58)	1311 (63)	1117 (62)	1214 (60)	1155 (63)	1152 (63)	Cond, size, cond*size
Movement time (ms)	710 (38)	585 (35)	990 (67)	716 (51)	828 (54)	886 (62)	Cond, size, cond*size
Deceleration time (ms)	550 (35)	429 (34)	812 (63)	546 (46)	646 (49)	709 (58)	Cond, size, cond*size
Response time (ms)	1154 (46)	1012 (43)	1483 (83)	1168 (60)	1325 (67)	1362 (76)	Cond, size, cond*size
Target error (mm)	3.4 (.31)	4.78 (.35)	4.52 (.37)	6.7 (.52)	3.63 (.31)	7.95 (.48)	Cond, size, cond*size

**Table 2:** Paired comparisons for the *Condition* factor showing the difference between unimanual (Uni), bimanual congruent (BiCon), and bimanual incongruent (BiInc) movements

Measure	Main effect	Comparison	*N*	Mean difference	SEM	*p*
Reaction time	*F* (2, 36) = 13.65, *p* < 0.001, $\eta_{p}^{2}$ = 0.431	Uni–BiCon	19	− 37.192	12.120	0.020
		Uni–BiInc	19	− 54.237	12.727	0.001
		BiCon–BiInc	19	− 17.045	5.411	0.017
Acceleration time	*F* (2, 36) = 10.51, *p* < 0.001, $\eta_{p}^{2}$ = 0.369	Uni–BiCon	19	− 16.016	4.942	0.014
		Uni–BiInc	19	− 18.808	5.424	0.008
		BiCon–BiInc	19	− 2.792	2.233	0.681
Peak speed	*F* (2, 36) = 42.61, *p* < 0.001, $\eta_{p}^{2}$ = 0.703	Uni–BiCon	19	111.627	17.961	< 0.001
		Uni–BiInc	19	123.887	16.481	< 0.001
		BiCon–BiInc	19	12.259	7.870	0.410
Movement time	*F* (2,36) = 58.82, *p* < 0.001, $\eta_{p}^{2}$ = 0.766	Uni–BiCon	19	− 205.941	26.446	< 0.001
		Uni–BiInc	19	− 209.514	25.951	< 0.001
		BiCon–BiInc	19	− 3.573	9.730	1.000
Deceleration time	*F* (2, 36) = 54.69, *p* < 0.001, $\eta_{p}^{2}$ = 0.752	Uni–BiCon	19	− 189.237	24.605	< 0.001
		Uni–BiInc	19	− 187.939	24.350	< 0.001
		BiCon–BiInc	19	1.297	10.113	1.000
Response time	*F* (2, 36) = 56.09, *p* < 0.001, $\eta_{p}^{2}$ = 0.757	Uni–BiCon	19	− 243	0.032	< 0.001
		Uni–BiInc	19	− 261	0.031	< 0.001
		BiCon–BiInc	19	− 018	0.015	0.732
Target error	*F* (2, 36) = 30.33, *p* < 0.001, $\eta_{p}^{2}$ = 0.628	Uni–BiCon	19	− 152	0.022	< 0.001
		Uni–BiInc	19	− 17	0.025	< 0.001
		BiCon–BiInc	19	− 018	0.025	0.479

**Table 3:** Simple effects resulting from the *Condition* x *Size* interactions that were found for limb movement measures

Measure	Condition x size	Condition		
		Unimanual	Bimanual congruent	Bimanual incongruent
Reaction time (ms)	*F* (2, 36) = 3.16, *p* = 0.05, $\eta_{p}^{2}$ = 0.149	*F* (1, 18) = 12.79, *p* = 0.002, $\eta_{p}^{2}$ = 0.415	*F* (1, 18) = 12.00, *p* = 0.003, $\eta_{p}^{2}$ = 0.400	*F* (1, 18) = 1.92, *p* = 0.19, $\eta_{p}^{2}$=0.096
Acceleration time (ms)	*F* (2, 36) = 0.34, *p* = 0.72, $\eta_{p}^{2}$ = 0.018	n/a	n/a	n/a
Peak speed (mm/s)	*F* (2, 36) = 17.85, *p* < 0.001, $\eta_{p}^{2}$ = 0.498	*F* (1, 18) = 40.77, *p* < 0.001, $\eta_{p}^{2}$ = 0.694	*F* (1, 18) = 39.58, *p* < 0.001, $\eta_{p}^{2}$ = 0.687	*F* (1, 18) = 0.09, *p* = 0.77, $\eta_{p}^{2}$ = 0.005
Movement time (ms)	*F* (2, 36) = 65.85, *p* < 0.001, $\eta_{p}^{2}$ = 0.785	*F* (1, 18) = 98.16, *p* < 0.001, $\eta_{p}^{2}$ = 0.845	*F* (1, 18) = 72.00, *p* < 0.001, $\eta_{p}^{2}$ = 0.800	*F* (1, 18) = 18.66, *p* < 0.001, $\eta_{p}^{2}$ = 0.509
Deceleration time (ms)	*F* (2, 36) = 65.94, *p* < 0.001, $\eta_{p}^{2}$ = 0.786	*F* (1, 18) = 106.27, *p* < 0.001, $\eta_{p}^{2}$ = 0.855 *F* (1, 18) = 106.27, *p* < 0.001,	*F* (1, 18) = 71.30, *p* < 0.001, $\eta_{p}^{2}$ = 0.798	*F* (1, 18) = 20.21, *p* < 0.001, $\eta_{p}^{2}$ = 0.529
Response time (ms)	*F* (2, 36) = 48.35, *p* < 0.001, $\eta_{p}^{2}$ = 0.729	*F* (1, 18) = 113.04, *p* < 0.001, $\eta_{p}^{2}$ = 0.863 *F* (1, 18) = 113.04, *p* < 0.001,	*F* (1, 18) = 61.47, *p* < 0.001, $\eta_{p}^{2}$ = 0.773	*F* (1, 18) = 4.05, *p* = 0.059, $\eta_{p}^{2}$ = 0.184

### Target error

**Fig. 3:**
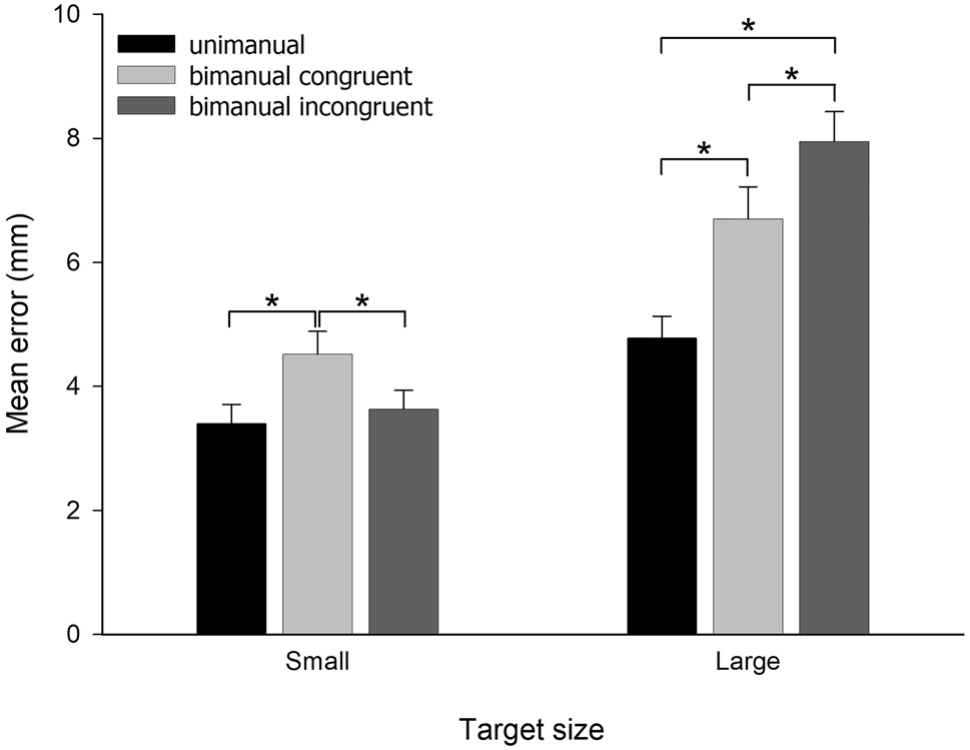
Mean target error as a function of Condition for each of the target sizes. Error bars denote the standard error of the mean. Asterisks denote statistical significance

Across all conditions, the dominant limb (0.49 mm) performed with less error than the non-dominant limb (0.55 mm) leading to a significant main effect of *Side*, *F* (1, 18) = 9.97, p = 0.005, $\eta_{p}^{2}$ = 0.357. There was also significant main effects of *Condition*, *F* (2, 36) = 30.33, p < 0.001, $\eta_{p}^{2}$ = 0.628, *Size*, *F* (1, 18) = 109.30, *p* < 0.001, $\eta_{p}^{2}$ = 0.859 and a *Condition* x *Size* interaction, *F* (2, 36) = 20.75, *p* < 0.001, $\eta_{p}^{2}$ = 0.535. The latter is best explained by referring to Fig. 3. As might be expected, participants deviated further from the centre of large targets (i.e., they showed greater error) than small targets, whatever the condition. In addition, bimanual movements resulted in greater overall error than unimanual movements. However, while the two bimanual conditions did not differ overall in terms of error, the pattern of errors was different. While the *cost* (i.e., increased error) of making congruent bimanual movements was borne by both limbs (*p* < 0.005 for small, *p* < 0.001 for large, both corrected), the cost of making incongruent bimanual movements was all borne by the limb moving to the large target; i.e., error for small targets was comparable for unimanual and bimanual incongruent conditions (*p* = 1.0. corrected).

### Limb coupling

**Fig. 4:**
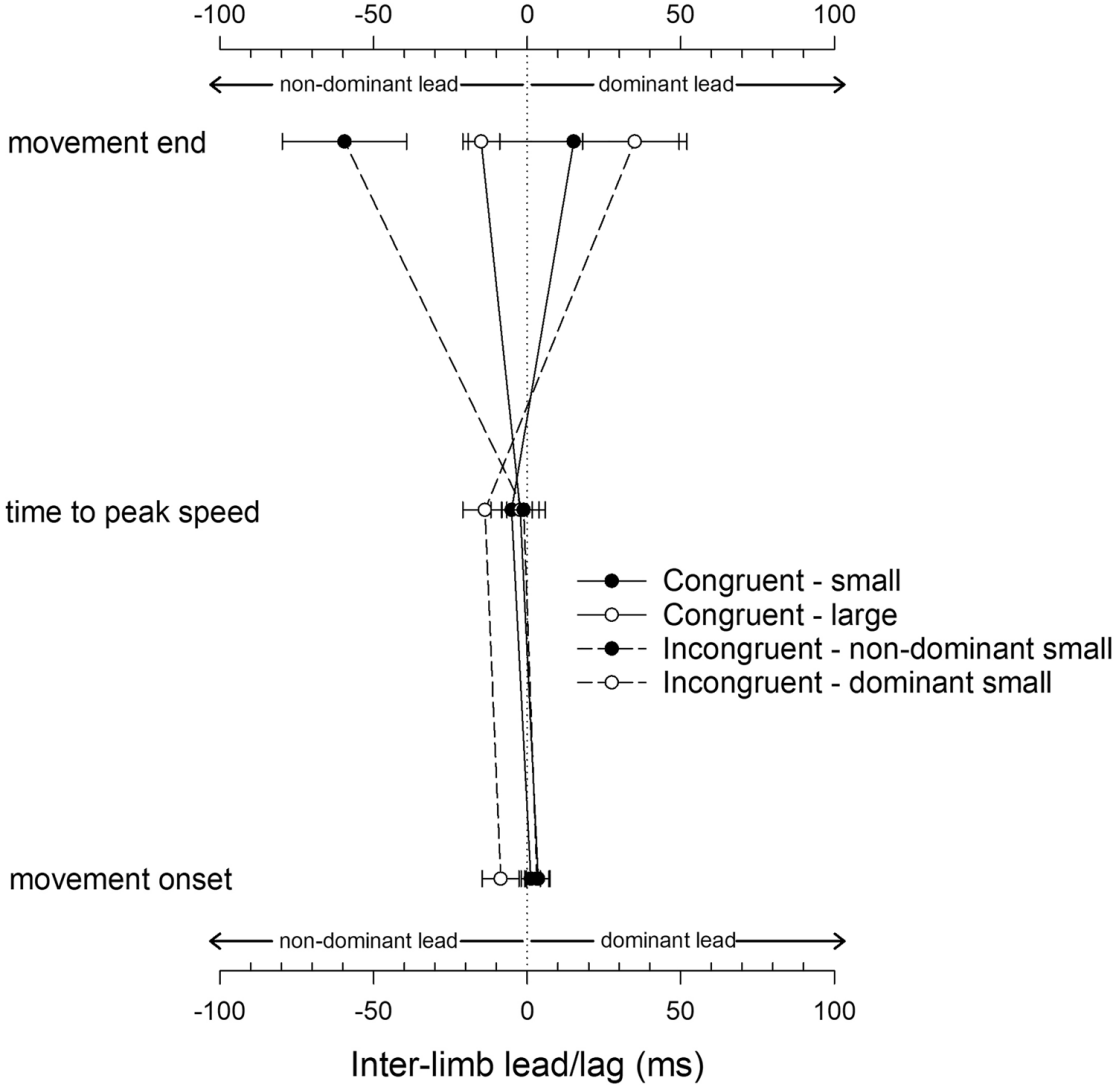
Inter-limb asynchrony (lead/lag) shown as a function of congruence and phase of movement. Error bars denote the standard error of the mean. Negative values indicate a non-dominant limb lead; positive value indicate a dominant limb lead

Figure 4 presents mean lag data reflecting temporal coordination between the limbs at movement onset, as the limbs reached peak speed and at movement end. For the first two of these measures, a 2 × 2 (Congruence x Dominant Target Side) ANOVA revealed no significant main effects and no interactions, supporting the strong coupling of bimanual movements. Indeed, mean values for all conditions were within 12 ms of zero (i.e., perfect synchrony) at these time-points. However, at movement end, asynchrony emerged and the ANOVA revealed an interaction, *F* (1, 18) = 11.45, *p* = 0.003, $\eta_{p}^{2}$ = 0.389. For congruent movements, the size of targets had no effect on coupling, *F* (1, 18) = 0.68, *p* = 0.42, $\eta_{p}^{2}$ = 0.036. However, for incongruent movements, there was a marked lead for the limb moving to the small target with a significant effect of Dominant Target Size, *F* (1, 18) = 10.36, *p* = 0.005 uncorrected, $\eta_{p}^{2}$ = 0.365.

The more pronounced asynchrony for incongruent bimanual movements at movement end was borne out by the *absolute* lag data. Here, the mean absolute lag was significantly larger for incongruent (106 ms) compared with congruent (67 ms) movements, *F* (1, 18) = 14.89, *p* = 0.001 uncorrected, $\eta_{p}^{2}$ = 0.453.

### Eye movements and relations with other measures

Time spent looking left and right was measured as a proportion of response time; accordingly, values of − 1 and + 1 indicate that the whole response time was spent looking towards the non-dominant and dominant sides, respectively. However, given that trials always began with participants fixating centrally, values never reached − 1 or + 1 but still provided a measure of bias in one direction or the other. For unimanual movements, participants typically made a single saccade in the direction of the single target. Mean values were − 0.72 and 0.68 for the non-dominant and dominant sides, respectively, resulting in a marked *Side* effect, *F* (1, 18) = 1820.99, *p* < 0.001, $\eta_{p}^{2}$ = 0.990. There was no effect of *Size* and no interaction.

**Fig. 5:**
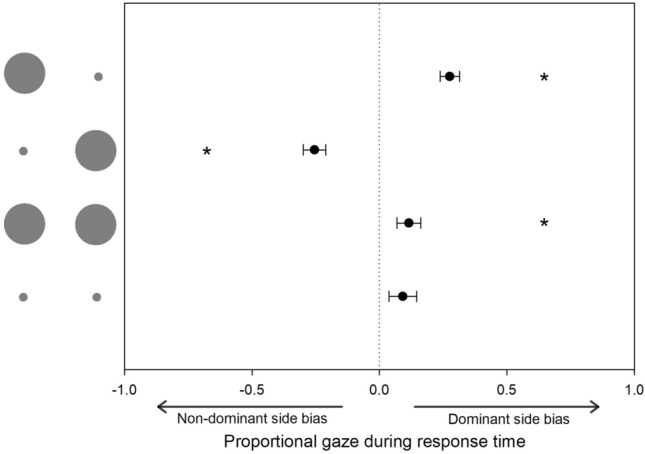
Direction of gaze as a proportion of total response time. Error bars denote the standard error of the mean. Negative values indicate gaze bias to the non-dominant side; positive values indicate a gaze bias to the dominant side. Asterisks denotes a statistically significant difference from the additive inverse

For bimanual movements, participants often made multiple saccades, but our measurement approach allowed biases to emerge (see Fig. 5). Our primary interest here was whether any lateralised bias was reliable. As a more conservative measure of this than simply comparing with zero, a comparison was conducted for each condition with the corresponding inverted data. For congruent conditions, although mean values were towards the dominant side, this was only reliable where targets were large (small, *t* (18) = 1.60, *p* = 0.13; large, *t* (18) = 2.60, *p* = 0.018). For incongruent targets, bias was far more pronounced. When the small target was on the dominant side, significantly more time was spent looking towards that side, *t* (18) = 6.32, *p* < 0.001. Similarly, there was a strong bias to the non-dominant side when the small targets was on that side, *t* (18) = − 5.92, *p* < 0.001.

**Fig. 6:**
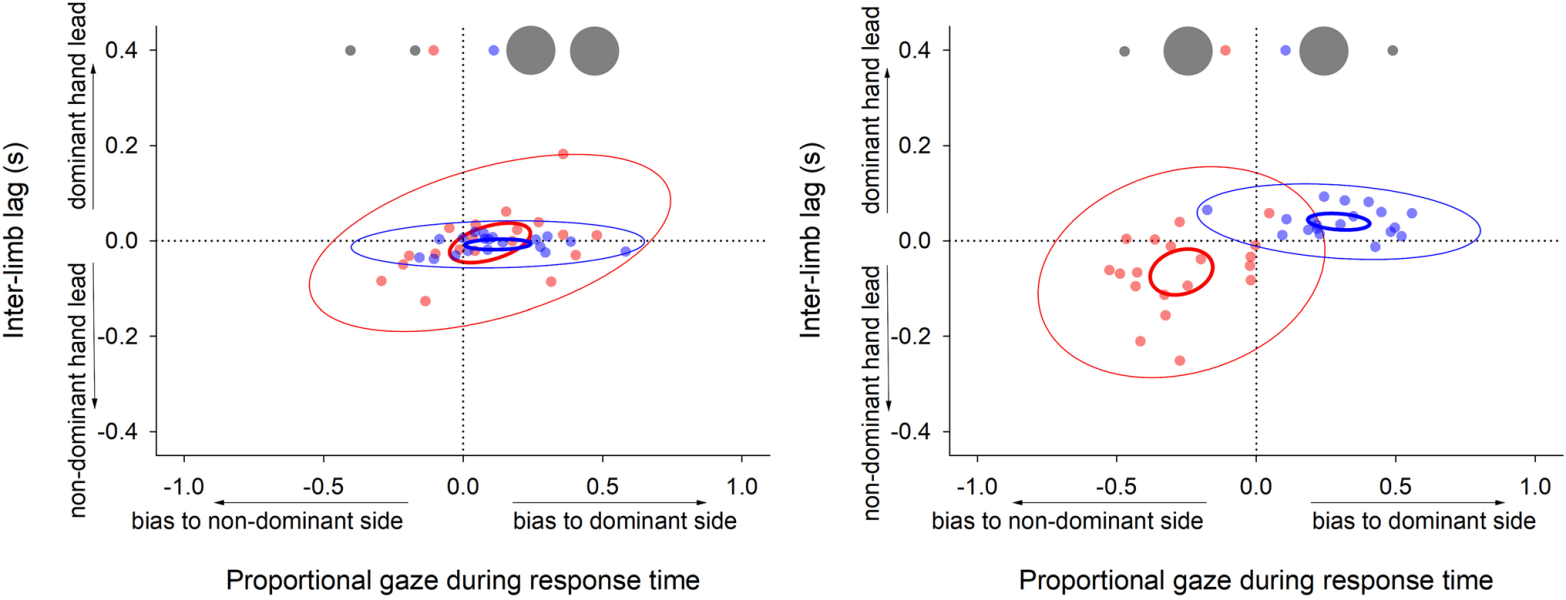
Scatterplots showing individual mean data as a function of proportional gaze bias during response time and inter-limb lag. Data are presented for both congruent (left panel) and incongruent (right panel) conditions and colour-coded for individual conditions. Ellipses denote confidence (thin line) and prediction (thick line) intervals

**Fig. 7:**
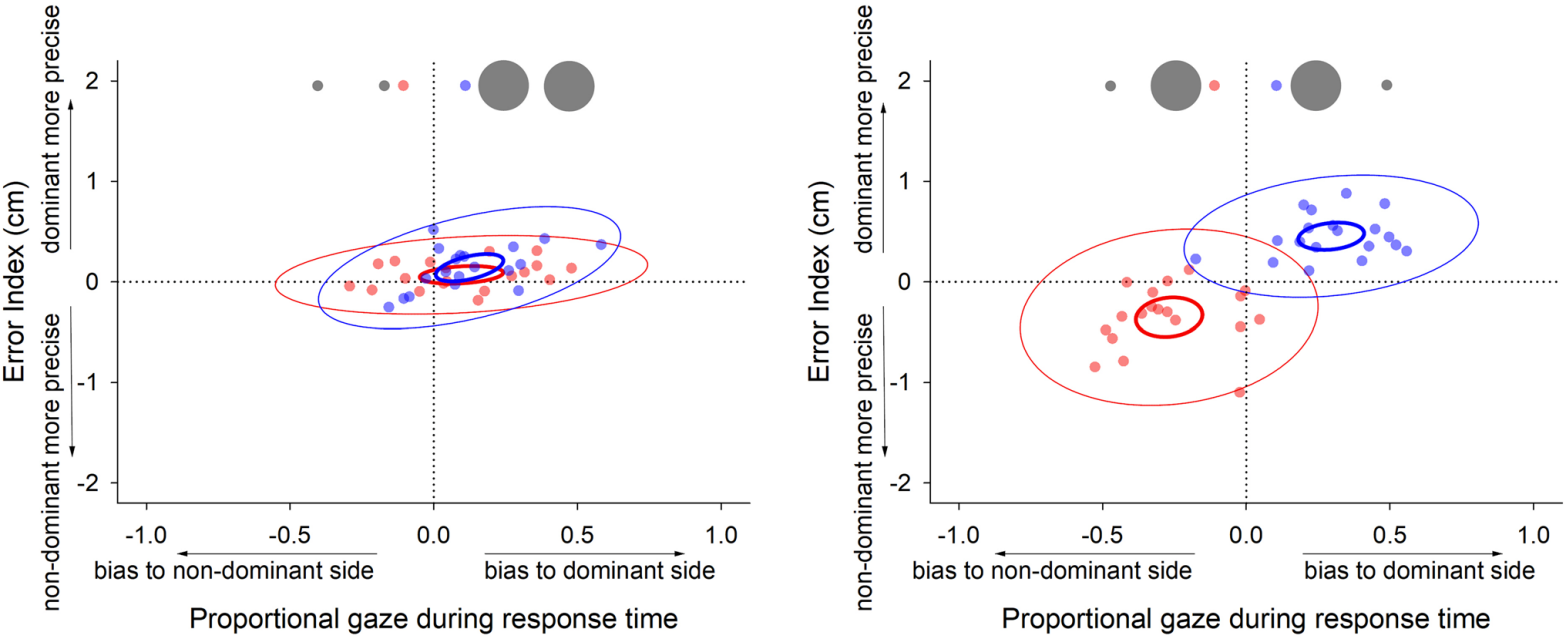
Scatterplots showing individual mean data as a function of proportional gaze bias during response time and error index (relative precision of each limb, calculated by subtracting target error for the dominant limb from the target error for the non-dominant limb). Data are presented for both congruent (left panel) and incongruent (right panel) conditions and colour-coded for individual conditions. Ellipses denote confidence (thin line) and prediction (thick line) intervals

Finally, to further highlight the orienting biases reported above and the accompanying inter-limb asynchrony also observed, Fig. 6 presents bivariate data for these two measures. Inspection of this figure underlines the pattern of data referred to above; where participants oriented more towards one side, the corresponding limb was the leading limb at movement end. Figure 7 shows the corresponding relationship between orienting bias and precision; the more precise limb movement was on the side to which visual orienting was biased.

## Discussion

This study set out to investigate the visual control of bimanual aiming movements. Participants made both unimanual and bimanual movements to targets which varied in size, while limb and eye movements were measured. For bimanual movements, targets presented were either the same size (termed *congruent*) or were of different sizes (termed *incongruent*). The basic aiming task used shared numerous similarities with previous studies of bimanual aiming and prehension, where kinematic data were reported in the absence of any eye movement data (Marteniuk et al. 1984; Corcos 1984; Fowler et al. 1991; Castiello et al. 1993; Jackson et al. 1999; Bingham et al. 2008; Bruyn and Mason 2009; Miller and Smyth 2012; Hesse et al. 2010). Before considering the eye movement data presented here and relations with the limb movement data reported, we first discuss how our limb kinematic data compare with these previous studies.

### Basic kinematic findings and similarities with previous studies

Kinematic data generated by the task demonstrated some well-established features of unimanual and bimanual control. First, unimanual movements to small targets were slower than those to large targets, and the IoD reflected in line with Fitts’ Law (Fitts 1954). Bimanual movements were also slower than unimanual movements, reflecting the *cost* of performing two movements simultaneously. This cost was also evident for reaction time confirming recent work highlighting the impact of visually guided bimanual movements on movement preparation (Blinch et al. 2018). Where bimanual movements were to equally sized targets (i.e., were *congruent*), Fitts’ Law was maintained with relatively slower movements when the targets were small. Also, consistent with previous studies (e.g., Jackson et al. 1999), where bimanual movements were to differently sized targets, Fitts’ law did not apply. Indeed, for these incongruent bimanual movements, while coupling between the limbs ensured that some measures (e.g., peak speed and response time) were comparable for movements to small and large targets, other measures (e.g., movement time, deceleration time) indicated faster movements for the *small* target. This provided the first indication in the present study that some asynchrony was occurring for bimanual movements (see later).

### Signs of asynchrony

Although average movement data are helpful to make some broad comparisons with the previous studies, it is important to recognise that these data can mask the asynchrony occurring on a trial-by-trial basis (Bruyn and Mason 2009; Miller and Smyth 2012). In this experiment, measures of both signed and absolute asynchrony derived from a trial-by-trial analysis revealed interesting findings.

At movement onset and during the early stages of movement, tight synchrony between the limbs was apparent and was consistent with the preponderance of influential bimanual coordination literature emphasising coupling between the limbs (Kelso et al. 1979; Swinnen 2002). However the asynchrony that emerged in the latter stages of movement and was captured at movement end appears to be driven by the varying visuo-motor requirements of the task and was largely consistent with a number of more recent studies (Miller and Smyth 2012; Bruyn and Mason 2009; Bingham et al. 2008).

Importantly, while these previous studies have tended to highlight the influence of the overall visuo-motor demands of the task in increasing asynchrony, here, it was the *relative* demands placed on each limb that was most critical in maximising asynchrony. Accordingly, asynchrony was most pronounced in the incongruent conditions rather than the most demanding condition (i.e., *congruent small*). It appears that the relative demands of each target create a competitive element typical of other visual attention tasks (Duncan et al. 1997) leading to prioritisation of the limb with the more demanding task and consequently greater asynchrony. Target error data were also supportive of this idea. For incongruent conditions, precision for small targets was comparable with that for unimanual movements (i.e., no noticeable cost of bimanual activity), while target error to the large target was greater. In contrast, both sides bore the cost of bimanual congruent movements equally.

### An initial consideration of eye movement data

Eye movement data during bimanual movements revealed that participants spent time looking towards each target for a given period of time on each trial and were therefore supportive of overt orienting being used to guide the movement of both limbs. When considering the proportion of time directing gaze to the dominant vs. non-dominant side, there was a tendency for more time to be spent directing vision towards the more difficult smaller target for incongruent trials, in a manner largely comparable with data reported by Riek et al. (2003).

Eye movement data were also partially consistent with a previous report highlighting a greater tendency to make eye movements towards the dominant side during bimanual aiming tasks (Honda 1982), e.g., there was a dominant-side bias in the congruent large condition. This finding is consistent with the general view that the dominant limb is more reliant on visual feedback for control (Goble and Brown 2008) with research suggesting a dominant-side attentional bias during bimanual tasks (Peters 1981; Buckingham and Carey 2009, 2014). However, a far more pronounced bias in overt orienting was observed during incongruent conditions. Here, regardless of the side (i.e., dominant or non-dominant), overt orienting was biased towards the more difficult (smaller) target.

### Relations between eye movements, asynchrony, and precision

Data here show a clear relationship between overt visual orienting and asynchrony during the latter stages of bimanual incongruent aiming movements. Visual orienting was biased towards the small target; and the limb reaching towards this target was also more likely to arrive at its target first, whether this was the dominant or the non-dominant limb. This limb also demonstrated greater precision. Importantly, increased asynchrony was not simply a function of difficulty; the condition with the highest index of difficulty was the bimanual congruent condition where both targets were small. Rather, the greatest asynchrony and the largest visual bias occurred when bimanual movements were made to targets with different IoDs. We propose that it is the competing demands of individual movements that drive greater desynchronization in related bimanual coordination. The situation where bimanual coordination involves component unimanual movements that have distinct indices of difficulty drives competition in the visuo-motor system. The challenge for the control system is to resolve the individual requirements of these movements while optimising the temporal coordination of both movements. Hence, one could see this as part of a continuum of bimanual control. At one end of this continuum, bimanual movements are perfectly synchronous, whereas at the other end, bimanual movements unfold serially. Factors contributing to the progressive desynchronization of bimanual movements appear to include (i) differing (i.e., incongruent) movement requirements of the limbs, (ii) the level of visual guidance required, (iii) manual asymmetry (i.e. dominance)—though not in this study, and presumably, (iv) impairment.

### Other considerations

It is possible that the precision requirements of this study were not so exacting as previous studies requiring the of movement of styli (Riek et al. 2003) and cylindrical objects (Srinivasan and Martin 2010) to the target locations. The more naturalistic pointing movements and the measuring of precision in the present study (i.e., error distance was measured in mm, rather than simply recording hit or miss) may account for these differences. Moreover, unlike these two previous studies, we found no evidence of a *hover phase*, described as a period where one limb remains stationary close to the target until both limbs are aligned before finally touching the targets together (Riek et al. 2003; Srinivasan and Martin 2010). Again, differences in task constraints are likely to account for these differences.

In this study, visual bias was represented by the relative amount of time participants spent looking left vs. right. While these data are informative, given the (sometimes) multiple saccades occurring during individual trials, it is important that future research explores the relationship between eye movements and temporal coupling between the limbs as bimanual movements unfold *within* a trial. It is possible that the leading limb switches multiple times within a trial, determined by the sequence of related eye movements taking place.

In terms of attentional orienting, this study tracked the direction of eye movements and was therefore primarily concerned with *overt* attentional orienting. It is important to acknowledge that previous studies have explored the control of bimanual reaching movements, while participants fixate their vision either centrally or on one particular target (Bruyn and Mason 2009; Jackson et al. 2002). In these studies, participants can only orient covertly and data suggest that the contribution of covert visual attention is not inconsiderable. For example, Bruyn and Mason (2009) showed that individuals accurately scale their grasp (a relative measure of precision) when performing bimanual reach to grasp movements to different-sized targets. This behaviour was similar whether participants were free to make eye movements or fixated their vision centrally between the two targets, suggesting good selective control using covert visual attention. However, as the acuity of visual information deteriorates rapidly with distance away from the point of foveation, it is not surprising that individuals select to make eye movements when performing bimanual movements in everyday life. Nevertheless, determining the relative contributions of *overt* vs *covert* visual orienting to the visual control of bimanual movements remains an area for future study.

As noted above, limb asynchrony appears to increase as a function of greater competition between target/objects. In the present experiment, the size of targets was manipulated, but greater competition could also be introduced by manipulating distance (e.g., Bruyn and Mason 2009) and the degree of separation between targets (e.g., Srinivasan and Martin 2010).

Finally, the visual control of bimanual movements is critical to normal everyday functioning and a major challenge for people who have impairments affecting their limb movements and/or vision. While previous studies have carefully investigated bimanual reaching movements in a range of different disorders, such as stroke (Jackson et al. 2000; Punt et al. 2005a, 2005b), Parkinson’s Disease (Castiello and Bennett 1997; Alberts et al. 1998), and spinal cord injury (Britten et al. 2018), we are not aware of any previous clinial studies that have investigated related visual control (i.e., eye movements) in these populations. Examining the visual control of bimanual movements in these populations, using approaches such as the one reported here would be informative and potentially allow coherent planning of rehabilitation strategies.

## Conclusion

In summary, this study provides further evidence of the complex and strategic manner by which humans control bimanual movements. It highlights the emphasis that recent research has placed on the asynchronies that can emerge as these movements unfold (Bruyn and Mason 2009; Miller and Smyth 2012; Srinivasan and Martin 2010). Target characteristics modulated the relative difficulty of the component unimanual movements. Performing these movements concurrently appeared to set up an element of competition, with individual movements competing for visual resources. Furthermore, where this resulted in a visual bias to one side, the related limb showed a strong tendency to reach its target first leading to asynchrony between the limbs during the latter stages of movement. Our data show how the visuo-motor system balances its apparent drive for synchrony in the planning and execution of bimanual movements with the need to visually guide the limbs to two different locations.

## Data Availability

The datasets generated during and/or analysed during the current study are available from the corresponding author on reasonable request.
